# Increasing gap in human height between rich and poor countries associated to their different intakes of N and P

**DOI:** 10.1038/s41598-017-17880-3

**Published:** 2017-12-15

**Authors:** Josep Peñuelas, Ivan A. Janssens, Philippe Ciais, Michael Obersteiner, Tamás Krisztin, Shilong Piao, Jordi Sardans

**Affiliations:** 1 CSIC, Global Ecology Unit CREAF-CEAB-UAB, Cerdanyola del Valles, 08193 Catalonia Spain; 2 CREAF, Cerdanyola del Valles, 08193 Catalonia Spain; 3 Research Group of Plant and Vegetation Ecology (PLECO), Department of Biology, University of Antwerp, B-2610 Wilrijk, Belgium; 4 Laboratoire des Sciences du Climat et de l’Environnement, IPSL, 91191 Gif-sur-Yvette, France; 5 International Institute for Applied Systems Analysis (IIASA), Ecosystems Services and Management, Schlossplatz 1, A-2361 Laxenburg, Austria; 6 Sino-French Institute for Earth System Science, College of Urban and Environmental Sciences, Peking University, Beijing, 100871 China

## Abstract

We analyzed mean height of men born in the 1960s, 1970s and 1980s in 80 countries. Both height and the change in height during the last decades were correlated with N and P intake, as well as the N:P intake ratio. Rich countries had higher per capita N and P intake than poor countries (on average 19.5 ± 0.3 versus 9.66 ± 0.18 kg N y^−1^ and 2.17 ± 0.04 versus 1.35 ± 0.02 kg P y^−1^), and also larger increases in per capita N intake (12.1 ± 2.0% vs. 7.0 ± 2.1%) and P intake (7.6 ± 1.0% vs 6.01 ± 0.7%), during the period 1961–2009. The increasing gap in height trends between rich and poor countries is associated with an increasing gap in nutrition, so a more varied diet with higher N, P, and N:P intake is a key factor to improve food intake quality in poor countries and thus shorten the gap with rich countries. More N and P are needed with the consequent requirements for a better management of the socioeconomic and environmental associated problems.

## Introduction

Height is a fundamental human trait. Within countries, taller people tend to have, on average, more education, higher earnings and possibly even higher social position^1–3^. They live longer and have a lower risk of cardiovascular and respiratory diseases, but a higher risk of some cancers (references in NCD-RISC, 2016^4^; Paajanen *et al*.^5^; World Cancer Research Fundation/American Institute for Cancer Research 2007^6^; Davies *et al*.^7^; Zhang *et al*.^8^).

Human height is a quantitative trait controlled by multiple genes^9,10^, but it also depends on environmental factors^10–13^. Among the latter, nutrition, particularly the intake of the main bio-elements, N and P, during childhood and adolescence, should be particularly important determinants of height during adulthood. Several studies have observed the link between infant growth and N-metabolism imbalances^14,15^ and with low P intake^16,17^. Moreover, the weight of infants at birth has been positively related to the P concentrations in blood of mothers during gestation^18^ and the growth rates of new-born infants have been linked to P concentration in mother’s milk^19^. Improvements in growth rates in infants have been associated to higher capacity to retain N in their body^20^.

The main source of N for humans comes from proteins and amino acids and at lesser extent from nucleic acids in food intakes^21,22^. Whereas in affluent countries the easy supply of N-fertilizers helps to produce an excess of food in general, and animal food in particular, in most underdeveloped countries the use of N-fertilizers makes the difference between adequate diet and malnutrition^22^. The main food sources of P are milk, chocolate and their derivatives, cereal grains, mollusks, beans and nuts, carbonate and cola beverages, fish and meat, and specially organ meats^23,24^. Most absorbed phosphorus comes from organic-P bound *in vivo* to animal and plant proteins although most plant phosphorus is mostly associated with phytates and is less absorbed in gut^23^. Several industrial processed food products include food additives rich in P^25^, which can even drive to an excess of P dietary intake that promotes higher serum parathyroid hormone (PTH) intake and lower serum Ca concentrations with negative impacts on bone health^25,26^.

In addition to the differences in total amount of food intake (calories), we hypothesized a greater gap in the per capita N and P food intake resulting from the higher per capita food intake from animal sources, richer in N, and the larger use of food additives, rich in P, in developed than in developing and undeveloped countries. We also hypothesized that this gap in the global N, P and the N:P intake ratio between developed and less developed countries is increasing and that it would go associated with an increasing gap in growth and adult height.

Here, we thus aimed to test the hypotheses that, across countries, i) there is a gap in the human height and the N, P and N:P intake ratio between developed and underdeveloped countries, II) this gap is increasing, and iii) the variability in human height and in recent trends therein is associated with the N and P contents (and their ratio N:P) in food intake and trends therein, while controlling for other factors which might influence height such as calories intake, total GDP, % of low weight infants at birth (less than 2500 g), the increase in urban population which might have an influence on average human height through an increase in pollution, and the Human Development Index (HDI) as indicators of economic, health and development conditions. HDI is a summary measure of average achievement in key dimensions of human development: a long and healthy life, being knowledgeable and have a decent standard of living. The HDI is the geometric mean of normalized indices for each of the three dimensions. The health dimension is assessed by life expectancy at birth, the education dimension by mean of years of schooling and the standard of living dimension by gross national income per capita.

To test these hypotheses, we used human height and food intake databases from FAO^27^, DTU^28^, USDA^29^, INFOODS^30^ and Universität Tübingen^31^, and analyzed the relationships of adult male height with per capita intake of N and P, the N:P ratio, food origin (animal or vegetal) and their changes in recent decades. We also considered the possible role of GDP, % of low weight infants at birth^32^, percentage of urban population, and per capita calories intake using databases from World Bank and FAO. HDI was obtained from Human Development Programme^33^.

**Figure 1:**
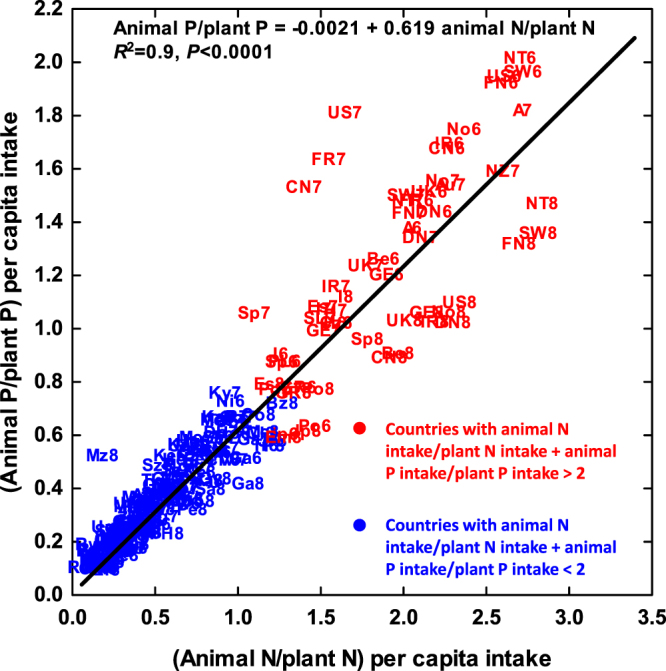
Relationships between (animal P/plant P) and (animal N/plant N) ratios per capita intake in different countries and human cohorts (1960s, 1970s and 1980s). The country abbreviations are: Armenia (Ar), Australia (A), Austria (Au), Azerbaijan (Az), Bangladesh (Ba), Belgium (Be), Benin (B), Bolivia (Bo), Botswana (Bt), Brazil (Bz), Burkina Faso (Bu), Cabo Verde (CV), Cambodia (Cm), Cameroon (Ca), Canada (CN), Central Africa Republic (Ct), Chad (CH), Chile (CI), China (C), Colombia (Co), Congo (Cg), Costa Rica (CR), Czechoslovakia (Cz), Denmark (DN), Djibouti (D), Dominican Republic (Do), Egypt (E), United Arab Emirates (Em), Estonia (Es), Ethiopia (Et), Finland (FN), France (FR), Gabon (Ga), Germany (GE), Ghana (Gh), Greece (GR), Guatemala (Gu), Guinea (Gi), Guyana (Gy), Haiti (Ha), Honduras (Ho), Hungary (H), India (In), Indonesia (Id), Iran (Ia), Ireland (IR), Italy (I), Jamaica (JA), Japan (Jp), Jordan (Jo), Kazahkstan (Ka), Kenya (Ke), North Korea (K), Kyrgyzstan (Ky), Lesotho (Le), Liberia (Li), Madagascar (Ma), Malawi (Mw), Mali (Ml), Mexico (Me), Moldavia (Mo), Morocco (Mr), Mozambique (Mz), Namibia (Na), Nepal (Ne), Netherlands (NT), Nicaragua (Ni), Niger (Ng), Nigeria (Nr), Norway (No), Panama (P), Peru (Pe), Phillipines (PH), Poland (PL), Portugal (Po), Russia (R), Rwanda (Rw), Saudi Arabia (As), Senegal (Se), Sierra Leone (Si), Slovenia (SL), South Africa (Sa), Spain (Sp), Sri Lanka (Sk), Swaziland (Sz), Sweden (SW), Taiwan (Tw), Tanzania (Ta), Togo (To), Trinidad Tobago (Tr), Tunisia (Tu), Turkey (Tk), Uganda (Ug) United Kingdom (UK), United States of America (US), Uzbekistan (Uz), Vietnam (VN), Yemen (Y), Zambia (Z), Zimbabwe (Zi). The countries with higher N and P intake from animal than vegetal products are indicated in red, and the countries with higher N and P intake from vegetal products are indicated in blue.

We defined two groups of countries to separate those with more animal-derived N and P intake from those with more plant-derived N and P intake, mostly coinciding with those with more N and P intake and those with less N and P intake respectively (Fig. 1): a) countries with (animal N/plant N) + (animal P/plant P) > 2 and those with this ratio <2. Figure 1 shows the relationship between (animal P/plant P) per capita intake versus (animal N/plant N) per capita intake in different countries and human cohorts (1960s, 1970s and 1980s).

## Results

**Figure 2:**
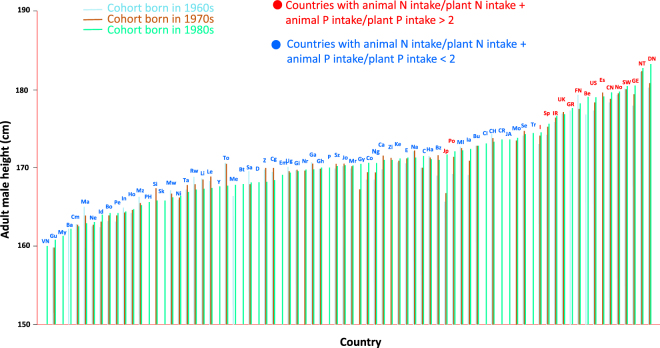
Mean heights of the men born in the 1960s (blue), 1970s (red) and 1980s (green) by country. The countries with higher N and P intake from animal than vegetal products are indicated in red, and the countries with higher N and P intake from vegetal products are indicated in blue. See the caption for Fig. 1 for the country abbreviations.

**Figure 3:**
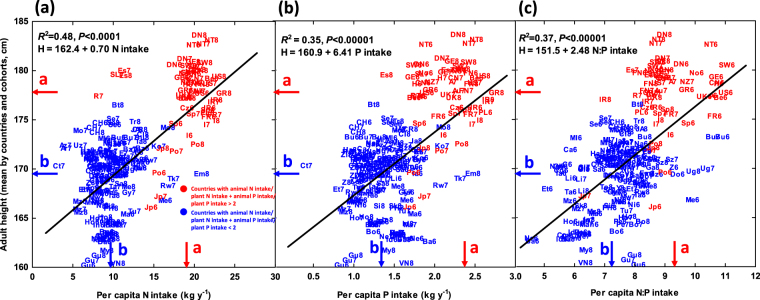
Relationships of mean male height with the average annual total N (**a**), P (**b**) and N:P ratio (**c**) intake per person (kg y^−1^) in the corresponding country and cohort. See the caption for Fig. 1 for the country abbreviations. Men born in the 1960s, 1970s and 1980s are identified by 6, 7 and 8 in the symbols, respectively. The countries with higher N and P intake from animal than vegetal products are indicated in red (also red arrow for the mean), and the countries with higher N and P intake from vegetal products are indicated in blue (also the arrow for the mean). Different letters on the axes indicate significant differences (*P* < 0.05). For detailed numerical values for each country and cohort see Table S2 (Supporting information).

**Figure 4:**
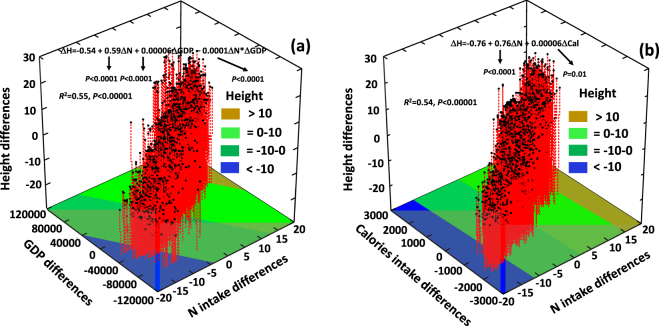
Relationships of mean male height differences (men cohort of 1980s) between all pairwise comparisons of the 80 countries studied with the corresponding differences in annual N intake and GDP (**a**), and with the corresponding differences in annual N intake and daily calories intake (**b**).

**Figure 5:**
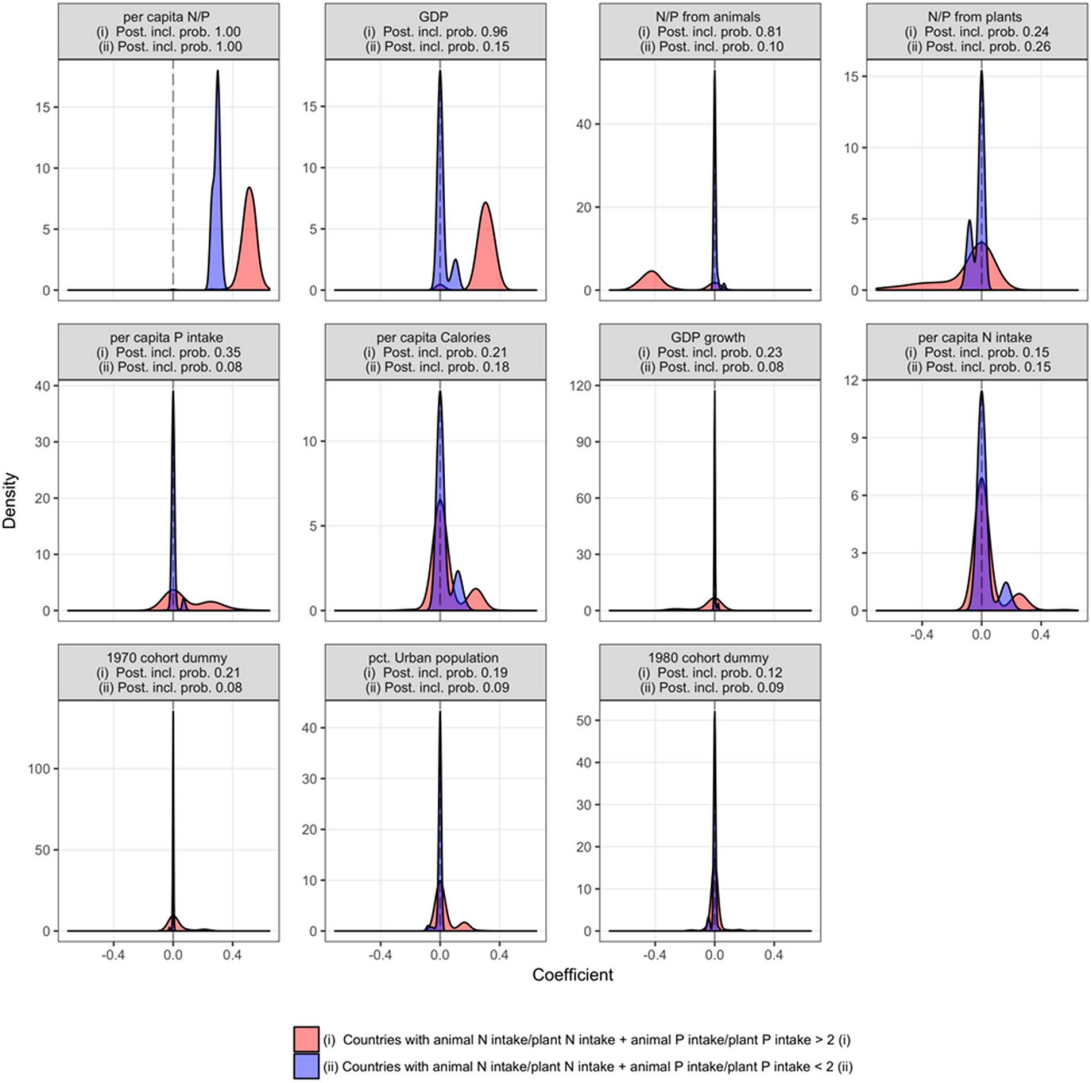
Posterior densities of the coefficient impacts for absolute human male height in the 1960s, 1970s and 1980s cohorts. The countries with higher N and P intake from animal than vegetal products are indicated in red (i), and the countries with higher N and P intake from vegetal products are indicated in blue (ii). The figures are ordered by the sum of the posterior inclusion probability of the displayed coefficients. A relative, high density mass at zero (signified by the vertical dashed line) corresponds to a high probability of the coefficient being excluded from the model.

Averaged over entire populations, men born in richer, higher-income countries had higher intake of animal than of vegetal products, had higher N, P and N:P intake, and were taller than men born in poorer countries (Figs 2–5). Across the 80 countries included in our analysis, the tallest adult men were found in Denmark and the Netherlands, ca. 183 cm, and the shortest were found in Guatemala and Vietnam, ca. 160 cm (Fig. 2).

Mean adult men height across different countries and human cohorts was correlated with per capita N intake, per capita P intake, N:P intake and Calories intake (R^2^ = 0.48, R^2^ = 0.35, R^2^ = 0.37 and R^2^ = 0.45, respectively) (Fig. 3, Figure S1a). The relationships of adult men height with GDP, HDI and % of low weight infants at birth were not linear. Men height increased strongly with increasing GDP at low values of GDP of poor countries and tends to progressively increase less when GDP reach values above 20000 $ per capita in developed richer countries (Figure S1b). The relationship of adult men height with HDI was positive only after HDI values around 0.4 mostly in developed countries (Figure S1c). Mean height strongly decreased with % of low weight infants at birth at the low values of this variable characteristic of developed countries but the relationship disappeared at the higher values characteristic of poorer countries (Figure S1d).

When analyzed by multiple regressions, the relationships between the absolute human male height differences among countries of 1980s cohort and the differences in N intake and GDP (Fig. 4a), the model explained 55% of total human male height differences, and total N intake had a stronger significance in the model (F = 521) than GDP (F = 82). Similarly, when considering the relationships between the absolute human male height differences among countries of 1980s cohort and the corresponding differences in per capita N intake and calories intake (Fig. 4b) the model explained a 54% of height differences, and total per N intake differences had a stronger significance in the model (F = 794) than the differences in calories intake (F = 82). The best SEM model explaining the relationships of height differences with differences in all the studied variables explained a 65% of height differences among countries (Figure S2). The SEM analysis showed once more that the relationships of height differences among countries with per capita N intake differences were stronger than with socioeconomic and sanitary variables such as GDP, % of low weight infants at birth and HDI differences. Moreover, the positive relationships of these socioeconomic variables with height differences among countries were mainly due to indirect effects through the effects on per capita N, P and N:P intake differences (Figure S2).

**Figure 6:**
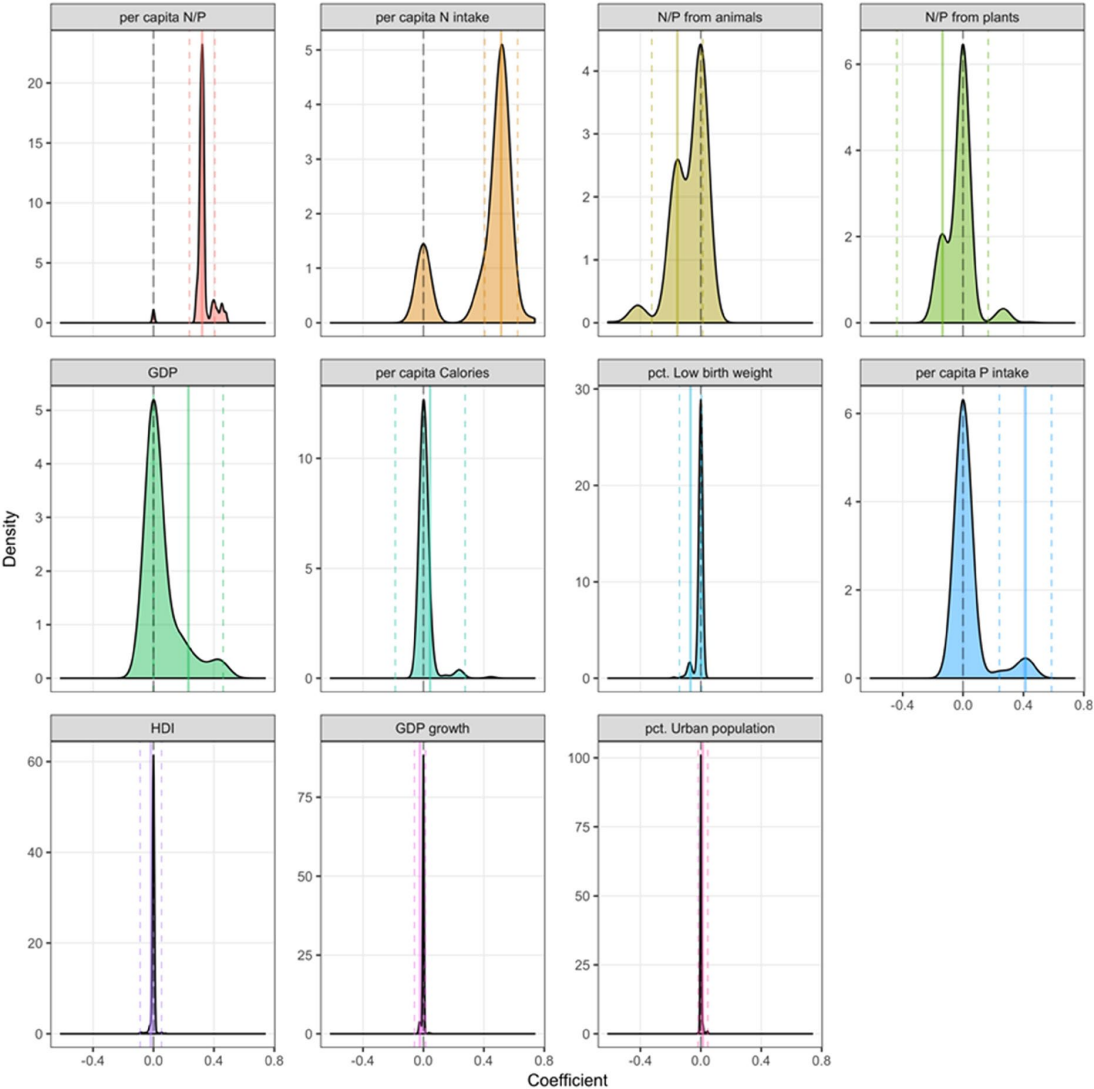
Posterior densities of the coefficient impacts for the differences between countries in the 1980s cohort, ordered by posterior inclusion probability. A high density mass at zero (signified by the vertical dashed line) corresponds to a high probability of the coefficient being excluded from the model. The bold, continuous colored lines denote the median posterior impact of the coefficient, conditional on its posterior inclusion probability. The dashed colored lines depict +/− two posterior standard deviations, conditional on the coefficient being included in the model.

Bayesian analyses of these data showed that the N:P ratio had the highest significant impact on human male height in poor and especially rich countries (Fig. 5). Rich countries also displayed a significant positive effect of GDP, whereas poor countries did not. The results of the Bayesian analyses considering also HDI and % of low weight infants at birth also reinforce that the most important factors for human height is the ratio of the per capita N and P intake (posterior inclusion probability of 97), as well as the per capita N intake (posterior inclusion probability of 81.6) (Table S5, Fig. 6).

**Figure 7:**
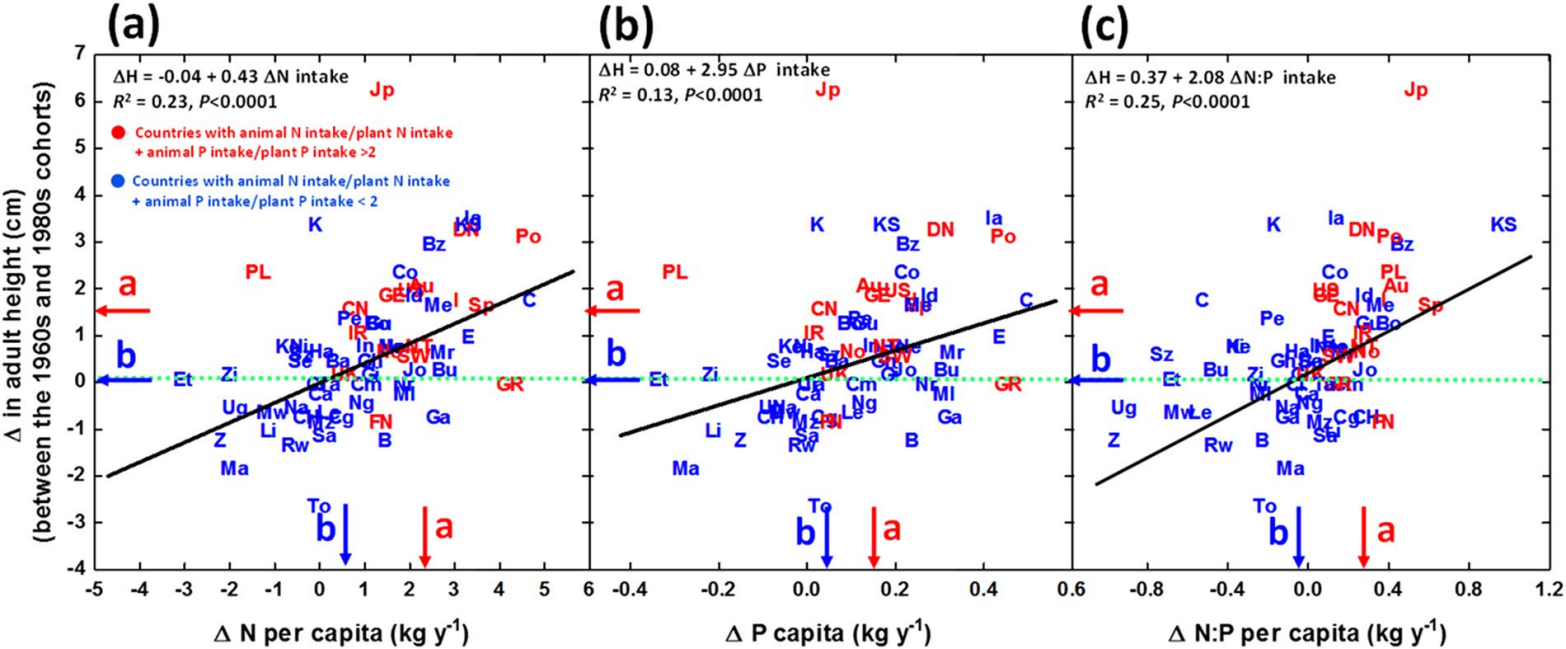
Relationships of the change in male height (in cm) from the 1960s cohort to the 1980s cohort in the countries with the increase in annual N (**a**), P (**b**) and N:P ratio (**c**) intake during the growth period (1960s-1980s for the 1960s cohort, and 1980s-2000s for the 1980s cohort). Red arrows represent the countries with higher N and P intake from animal products, and blue arrows represent the countries with higher N and P intake from vegetal products. Different letters on the axes indicate significant differences (*P* < 0.05). See the caption for Fig. 1 for the country abbreviations.

Relative to men born in the 1960s, the largest increases in adult height in men born in the 1980s were found in Japan, ca. 6 cm, and Denmark and Portugal, ca. 3 cm (Fig. 7). In contrast, adult height decreased in some countries, mainly from the African continent, with the largest decreases in Togo, ca. -3 cm, followed by Madagascar, ca. −2 cm. Height changes differed greatly between rich and poor countries, with an average increase in height in rich countries of 1.5 cm in the cohort born in the 1980s relative to the cohort born in the 1960s, and with no increase in average height across the poor countries during the same period (Fig. 7).

Although less sensitive (lower slopes in the regression lines) than the absolute height, also the change in height between men born in the 1960s and in the 1980s was correlated with the change in N and P intake during the same period (Fig. 7). Rich countries increased the intake of N (on average 2.3 ± 0.4 kg y^−1^, i.e. 12.1 ± 2.0%) more than the intake of P (on average 0.17 ± 0.03 kg y^−1^, i.e. 7.6 ± 1.0%), so the intake N:P ratio or imbalance between N and P has increased (0.3 ± 0.08, i.e. 3.53 ± 0.73%). Poor countries increased significantly less their intake of N (0.7 ± 0.2 kg y^−1^, i.e. 7.0 ± 2.1%) or P (0.08 ± 0.2 kg y^−1^, i.e. 6.01 ± 0.7%), and their N:P ratio did not change statistically significantly. These minor increases in nutrient intakes occurred in parallel with a lack of an overall increase in height, with many individual countries, mostly African, even exhibiting a decreasing trend in height (Fig. 7).

Differences in in N:P, P and N intake had the largest posterior impact on differences in human height between cohorts (Figure S4). Based on the densities, we can reject the hypothesis, that – when controlling for N and P intake – the differences in calories or urban population have an impact that is significantly different from zero on the differences of human height. The results of the Bayesian analyses including also HDI and % of low weight infants at birth also reinforced the main role of N:P and N and P intake (Figure S4).

## Discussion

Average male height was highly variable among countries, with a difference of ca. 23 cm between the tallest and shortest country means. This difference was associated with differences in the per capita N, P and N:P intake among countries, more strongly than with other possible explanatory factors such as daily calories intake associated to energy for living but not directly to building blocks for growth.

In recent decades, adult height has changed substantially in many countries. The change has been substantial but less sensitive to the recent change in N and P intake than expected from the general relationships between height and N and P intake (lower slopes in the regression lines). This slower response to changes in N and P intake at short term indicates the existence of other scaling factors integrated at longer time scales in the relationship height-N and P intake. Genetic and epigenetic changes are involved at these longer term scales. Genetic variation explains the reduced slope of the relationship between increased intake of nutrition factors and differences in height between time intervals: there is a limit to the phenotypic response to improved environmental conditions that a fixed genetic set up can produce. The increasing differences in immigration rates among countries, though, will generate different levels of gene flow. This gene flow can introduce new variation that can change the phenotypic response in the new cohorts.

Although height trends varied strongly among countries, the difference in adult male height between the rich and poor countries increased, on average, by ca. 1.5 cm in the last three decades. This increasing height difference was the result of men in richer countries becoming even taller (on average), whereas the men in poor countries did not increase in height (on average). This divergent height growth pattern was associated with a different trend in N and P intake between rich and poor countries, and specially with increasing N:P ratio of food intake, indicative of increasingly larger relative intake of food of animal origin in rich countries, and indicative of absence of saturation in height and in N and P intake. Food of animal origin has, on average, higher N and P contents and N:P ratios than food from plants, and the percentage of animal-derived food of total intake is higher in developed than less developed countries (Fig. 1). In addition to N and P intake and nutrition, other factors may impact on the increase of height as highlighted by the very high increase in Japan, much above the explained value by the overall relationships with N, P and N:P uptake (Figs 3 and 7).

Our results, both of current mean height and its changes in recent decades, are consistent with available data and trends previously recorded in many countries, usually treated as indicators of social and environmental conditions that affect nutrition, health and economic prosperity in each country and generation. For example, previous studies have documented an increase in stunting in men in some poor countries, such as those of sub-Saharan Africa^4,34^. Rising population, coupled with worsening economic status, may underlie the decrease in height in these African countries^34–37^.

Height is not a neutral trait. In addition to behavioral and social links, higher adult height of men within the population of a same country or geopolitical region is known to be associated with lower occurrence of cardiovascular and respiratory diseases, but also with higher occurrence of colorectal and possibly pancreatic and prostate cancers, independently of its inverse correlation with body mass index^6,7,38^. If these associations would be causal^39^, then the ca. 23 cm height range globally could be associated with a 17% lower risk of cardiovascular mortality and 20–40% higher risk of various site-specific cancers in countries with tall people relative to countries with short people^40^. Increases in mean population height in successive cohorts are associated with lower mortality in middle and older ages in countries with reliable mortality data^40^, consistent with individual-level evidence of the association between taller height and lower mortality from all causes in adults^41^. Caution must, however, clearly drive the application of these associations when comparing among countries and geopolitical regions instead of individuals of the same population group.

The average height of young adult men in different countries is also an indicator of sustainable human development^40^. Adult height reflects not only fetal and early childhood nutrition, which was included in the Millennium Development Goals, but also that of adolescents^34^. Adult height is also associated with these early-life experiences and with longevity, education and earnings. It can easily be measured in health surveys and can be used to investigate differences across countries and trends over time, as we have, and to investigate within-country inequalities.

The association between height and nutrition, particularly with N and P intakes and the corresponding N:P ratio, highlights the importance of these two bio-elements. The increases in N and P intake in rich countries favor rapid protein synthesis and maximizes the genetic capacity for attaining a maximal height. Several other factors in which rich and poor countries differ, such as the quality and accessibility of sanitary services, can indirectly influence the differences in height, but it is food that provides the main sources, the building blocks, to grow while other factors can directly influence more other variables such as life expectancy and health. More N and P intake is a direct consequence of the intake of more proteins, which are the molecules underlying growth processes at cellular scale. The relationship between socioeconomic variables and the gap of human height between rich and poor countries is mostly due to their indirect effects through total per capita N and P intake and N:P intake ratio.

The increase in height is thus associated to larger N and P intakes. For N-rich crops N fertilization may not be a problem, but most soils globally contain only low concentrations of available forms of P, so P inputs in the form of fertilizers are essential for food production around the globe. The majority of agricultural P is mined from finite and therefore exhaustible^42–45^ sedimentary deposits of phosphorite. The growing human and livestock populations requiring food and feed, and the growing demand for biofuels may exhaust these reserves in the next 40 to 400 years^42–44^. P is thus expected to become economically inaccessible to low-income and food-deficient countries, which will likely further widen the gap in nutrition and therefore in height and health between rich and poor countries. The implications of this yield gap to food security add a key additional requirement of fundamental changes in agricultural and food management to those already posed by the need of mitigating climate change.

Our results show that increases in N and P intakes are more important than the increases in calories intake to increase height in a given country. Thus, a more varied diet with higher N and P intake is a key factor to improve food quality in poor countries and thus shorten the gap with rich countries. The range of calories intake between countries (from 1879 to 3721 daily Cal intake) is much narrower than the differences in N (from 3.3 to 23.7 per capita kg y^−1^) and P intake (from 0.41 to 2.76 per capita kg y^−1^), meaning that not only calories intake must be increased but mainly N and P intake and food variability (more proteins and vitamins). This requires increasing crops with more N and P content, and/or animal derived food production, which by itself has severe sustainable drawbacks^46^. Consequently more sources of N and P will be needed to improve the food quality and variability of poor countries with the consequent socioeconomic and environmental problems to be solved.

## Materials and Methods

We estimated the mean height of young adults born in the 1960s, 1970s and 1980s (i.e. people who had reached their 21st birthday between 1981 and 2010) in 80 countries. We used the data from University of Tubingen-World Height databases (www.uni-tuebingen.de) that provided the average adult male heights for each of these three decades. This database contains all data available from official sources including WHO/UNESCO, U.S. Department of Health and Eurobarometer, among others. We organized countries based on per capita N and P intakes of animal and plant origin (Supplementary Table 1).

We calculated the annual food intake of N and P per person for the three cohorts with data of adult height for each country as:

(∑ annual intake of each food group^[1]^ × mean N or P concentration of each food group^[2]^)/number of years

- Data from FAOSTAT (2014)

- Data from INFOODS_FOOD_DATABASE FOR BIODIVERSITY (2016), USDA (2016) and DTU Fodevareinstituttet (2016).

To estimate the N and P concentrations of each food group of FAO databases we used the data bases [2]. In these data bases there are the N and P concentrations of the different food items. We grouped these different food items in different sets corresponding to the distinct FAO food groups, and made the corresponding average of each of these groups. We used the average as a final value when data for N and/or P concentrations were provided by more than one database for the same food group.

To obtain the mean annual intake of N and P per person in each country during the period of growth of the three human cohorts with data for male height, we used the average from 1961 to1989 for the cohort born in the 1960s, from 1971 to 1999 for the cohort born in the 1970s and from 1981 to 2009N for the cohort born in the 1980s. The N:P ratios were estimated on a mass basis.

The increases in annual P and N intake and adult male height for each country in men born in the 1980s relative to those born in the 1960s were estimated for all countries and cohorts for which information was available.

We obtained the data of GDP and percentage of urban population from World Bank^47^. To obtain data of daily per capita calories intake we used data from FAO (http://www.fao.org/economics/ess/food-security-statistics/food-security-statistics-metadata/en).

We used reduced major axis analysis to assess the relationships of height and height change with N, P and N:P ratio intake for all countries and territories. We used general linear models to analyze the relationship of men height differences between all the pairwise comparisons among the studied countries with the corresponding differences in per capita calories intake, GDP and per capita N and P annual intake. We also conducted general linear models to analyze the relationships between the country men height with the corresponding country GDP, and per capita daily calories, N, P and N:P intakes. We similarly analyzed the relationships of the differences in men adult height between the cohort of the 1980s and the cohort of the 1960s with the changes in GDP, per capita calories, N, P and N:P intake during their respective growth periods.

Due to the limited number of observations and the lack of a strictly theoretical framework, we also used a Bayesian model averaging^48^ approach to explore a large number of potential models and draw inference over the relative importance of individual covariates. Moreover, the resulting parameter estimates stem from averages over the models with the highest posterior probability, thus providing robust estimates.

We modeled human height in absolute levels and across the cohorts with a Bayesian model averaging approach. For this purpose we had 208 observations, ranging over 86 countries. The average male human height in a country *i* in cohort $t$ was modeled as:

1 $$\begin{array}{rcl}heigh{t}_{it} & = & \sum _{k=1}^{2}[{\beta }_{1}+{\propto }_{t}+{\beta }_{2}{N}_{it}+{\beta }_{3}{P}_{it}+{\beta }_{4}GD{P}_{it}+{\beta }_{5}GDP{g}_{it}\\  &  & +\,{\beta }_{6}Ca{l}_{it}+{\beta }_{7}{N}_{it}\\  &  & +\,{\beta }_{8}{P}_{it}+{\beta }_{9}{\frac{N}{P}}_{it}\\  &  & +\,{\beta }_{10}{P}_{animal}/{P}_{vegetable{s}_{it}}+{\beta }_{11}{N}_{animal}/{N}_{vegetable{s}_{it}}\\  &  & +\,{\beta }_{12}Urba{n}_{it}]{I}_{({\omega }_{itk}=1)}+{\varepsilon }_{it}\end{array}$$

where *height*_*it*_ denotes the height of the *t*-th cohort in country *i*. As in the previous equation, *β*_1_ is the intercept, ∝_t_ a period specific dummy, *N*_*it*_ and *P*_*it*_ are the levels of N and P intake per capita in cohort t *GDP*_*it*_, *GDPg*_*it*_, *Cal*_*it*_, and *Urban*_*it*_ denote the level of GDP, GDP growth, calories and percentage of urban population, respectively. The ratios of N and P intake, as well as N and P intake from animals in proportion to vegetables are denoted by ${P}_{animal}/{P}_{vegetable{s}_{it}}$, and ${N}_{animal}/{N}_{vegetable{s}_{it}}$, respectively. *β*_2_ to *β*_12_ are the corresponding coefficients, and *ε*_*it*_ is the i.i.d. normally distributed error term with zero mean and *σ* ^2^ variance. *I*_(·)_ denotes an indicator function, which takes on the value of one if the condition *ω*_*itk*_ = 1 is fulfilled and is zero otherwise. *ω*_*it*1_ takes on the value of one if the sum of the N intake from animals per the N intake from vegetables plus the P intake from animals per the P intake from vegetables exceeds two and is zero otherwise. In this fashion we can model the impacts of both groups in one joint model, with a joint variance function.

We had complete data for the three cohorts for 54 countries in total. Taking the differences between the cohorts, results in *n* = 108 observations. We analysed these data with a similar Bayesian model where *i* denotes a specific country (with *i* = 1, …, *n*), and *t* the specific cohort:

2 $$\begin{array}{rcl}{\rm{\Delta }}{(height)}_{it} & = & {\beta }_{1}+{\propto }_{t}+{\beta }_{2}{N}_{it}+{\beta }_{3}{P}_{it}+{\beta }_{4}{\rm{\Delta }}{(GDP)}_{it}\\  &  & +\,{\beta }_{5}{\rm{\Delta }}{(GDPg)}_{it}+{\beta }_{6}{\rm{\Delta }}{(Cal)}_{it}\\  &  & +\,{\beta }_{7}{\rm{\Delta }}{(N)}_{it}+{\beta }_{8}{\rm{\Delta }}{(P)}_{it}+{\beta }_{9}{\rm{\Delta }}{(\frac{N}{P})}_{it}\\  &  & +\,{\beta }_{10}{\rm{\Delta }}{({P}_{animal}/{P}_{vegetables})}_{it}+{\beta }_{11}{\rm{\Delta }}{({N}_{animal}/{N}_{vegetables})}_{it}\\  &  & +\,{\beta }_{12}{\rm{\Delta }}{(Urban)}_{it}+{\varepsilon }_{it}\end{array}$$

where the ∆(∙) denotes the differences between cohorts *t* and *t* + 1. Δ(*height*)_*it*_ denotes the difference in height between cohorts. *β*_1_ is the intercept, ∝_t_ a period specific dummy, *N*_*it*_ and *P*_*it*_ are the levels of N and P intake per capita in cohort t Δ(*GDP*)_*it*_, Δ(*GDPg*)_*it*_, Δ(*Cal*)_*it*_, Δ(*N*)_*it*_, Δ(*P*)_*it*_ and ${\rm{\Delta }}{(Urban)}_{it}$ denote the difference in GDP, GDP growth, calories, N intake, P intake and percentage of urban population, respectively. The differences in ratios of N and P intake, as well as N and P intake from animals in proportion to vegetables are denoted by ${\rm{\Delta }}{({P}_{animal}/{P}_{vegetables})}_{it}$, and ${\rm{\Delta }}{({N}_{animal}/{N}_{vegetables})}_{it}$, respectively. *β*_2_ to *β*_12_ are the corresponding coefficients, and *ε*_*it*_ is the i.i.d. normally distributed error term with zero mean and *σ* ^2^ variance.

We finally also conducted these same Bayesian analyses for those countries of the data base for which we had these same variables and additional variables HDI and % of low weight infants at birth.

## Electronic supplementary material

Supplementary Information
