# Supplementary material for: Increasing gap in human height between rich and poor countries associated to their different intakes of N and P
Source: Sci Rep. 2017 Dec 15;7:17671. doi: 10.1038/s41598-017-17880-3 (PMC5732171; doi:10.1038/s41598-017-17880-3)
Supplement: Supplementary file 1 — Supplementary Information [file 41598_2017_17880_MOESM1_ESM.pdf]

Supplementary material: Increasing gap in human height between rich and poor countries associated to their different intakes of N and P.

Josep Peñuelas[1,2], Ivan A. Janssens[3], Philippe Ciais[4], Michael Obersteiner[5], Tamás Krisztin[5], Shilong Piao[6], Jordi Sardans[1,2]

[1] CSIC, Global Ecology Unit CREAF-CEAB-UAB, Cerdanyola del Vallès, 08193 Catalonia, Spain.

[2] CREAF, Cerdanyola del Vallès, 08193 Catalonia, Spain.

[3] Research Group of Plant and Vegetation Ecology (PLECO), Department of Biology, University of Antwerp, B-2610 Wilrijk, Belgium.

[4] Laboratoire des Sciences du Climat et de l'Environnement, IPSL, 91191 Gif-sur-Yvette, France.

[5] International Institute for Applied Systems Analysis (IIASA),

Ecosystems Services and Management, Schlossplatz 1, A-2361 Laxenburg, Austria.

[6]Sino-French Institute for Earth System Science, College of Urban and Environmental Sciences, Peking University, Beijing 100871, China.

*Correspondence to: josep.penuelas@uab.cat

Table S1. Average annual N and P intake from animal and plant sources and animal/plant ratio of N and P intake during the period 1980-2009 (coinciding with the growth period of the 1980s cohort). We established two groups of countries those with: N (animal/plant) + P (animal/plant) > 2 and those with: N (animal/plant) + P (animal/plant) < 2, to separate the countries with most N and P intake coming from animal food sources from those most N and P intake coming from plant food sources. In bold type are highlighted the countries with: N (animal/plant) + P (animal/plant) > 2.

Country	Annual N intake (kg) from animals	Annual N intake (kg) from plants	N (animal/plant)	Annual P intake (kg) from animals	Annual P intake (kg) from plants	P (animal/plant)
Bangladesh	1.27	7.73	0.165	0.124	1.43	0.086
**Belgium**	**13.7**	**7.00**	**1.96**	**1.30**	**1.01**	**1.29**
Bolivia	4.37	5.79	0.754	0.357	0.872	0.409
Botswana	4.87	5.68	0.857	0.539	0.766	0.703
Brazil	7.51	5.94	1.26	0.708	0.906	0.782
Burkina	1.72	11.7	0.147	0.158	1.13	0.139
Cabo Verde	4.85	5.39	0.900	0.492	0.834	0.589
Cambodia	1.95	7.87	0.248	0.150	1.50	0.100
Cameroon	2.11	6.35	0.332	0.179	0.892	0.200
**Canada**	**13.4**	**7.01**	**1.91**	**1.30**	**0.957**	**1.35**
Chad	2.05	5.73	0.356	0.194	0.844	0.230
Chile	7.20	6.38	1.13	0.678	0.940	0.721
China	3.95	8.27	0.478	0.299	1.38	0.216
Colombia	8.18	6.91	1.18	0.641	1.08	0.591
Congo	5.90	5.25	1.12	0.613	0.842	0.728

**Denmark**	**14.1**	**6.12**	**2.30**	**1.33**	**0.915**	**1.45**
Djibouti	2.96	4.19	0.707	0.326	0.680	0.479
Egypt	3.31	9.61	0.344	0.308	1.46	0.210
**Finland**	**15.3**	**5.68**	**2.70**	**1.61**	**0.850**	**1.90**
Gabon	6.75	6.41	1.05	0.525	1.01	0.520
**Germany**	**13.4**	**6.27**	**2.14**	**1.28**	**0.924**	**1.38**
Ghana	2.36	6.06	0.390	0.188	0.974	0.193
**Greece**	**13.2**	**9.86**	**1.34**	**1.30**	**1.40**	**0.924**
Guatemala	2.99	5.13	0.582	0.287	0.727	0.394
Guinea	1.32	7.55	0.175	0.118	1.29	0.092
Guyana	6.08	7.03	0.865	0.578	1.21	0.477
Haiti	1.43	5.46	0.261	0.127	0.818	0.155
Honduras	4.39	4.49	0.976	0.476	0.657	0.723
India	2.08	7.14	0.292	0.248	1.17	0.212
Indonesia	1.94	8.31	0.234	0.156	1.51	0.103
Iran	3.82	9.16	0.417	0.364	1.36	0.268
**Ireland**	**15.6**	**7.12**	**2.19**	**1.61**	**1.05**	**1.53**
**Italy**	**13.9**	**8.42**	**1.65**	**1.33**	**1.19**	**1.11**
Jamaica	7.07	5.84	1.21	0.663	0.905	0.732
**Japan**	**9.48**	**6.72**	**1.41**	**0.826**	**1.05**	**0.785**
Jordan	4.76	6.83	0.697	0.458	0.997	0.459
Kenya	3.89	5.07	0.767	0.451	0.720	0.626
Lesotho	2.00	6.56	0.305	0.179	0.963	0.186
Liberia	1.36	6.49	0.209	0.104	1.15	0.091
Madagascar	2.79	7.02	0.397	0.269	1.28	0.210
Malawi	0.961	6.01	0.160	0.081	0.852	0.095
Mali	3.69	6.92	0.533	0.389	1.12	0.348
Mexico	7.25	6.31	1.15	0.696	0.895	0.777
Morocco	2.93	8.59	0.341	0.257	1.24	0.207
Mozambique	0.861	4.78	0.180	0.071	0.778	0.091
Myanmar	2.32	6.49	0.358	0.192	1.18	0.162
Namibia	4.25	5.24	0.811	0.405	0.815	0.496
Nepal	1.96	7.46	0.264	0.211	1.28	0.165
**Netherlands**	**16.1**	**5.67**	**2.83**	**1.66**	**0.813**	**2.04**
Nicaragua	3.24	4.59	0.705	0.336	0.706	0.476
Niger	2.93	6.85	0.428	0.287	1.10	0.262
Nigeria	1.54	7.99	0.192	0.128	1.10	0.116
Norway	**14.1**	**6.20**	**2.27**	**1.42**	**0.907**	**1.57**
Panama	5.98	4.91	1.22	0.531	0.800	0.663
Peru	4.11	5.71	0.720	0.401	0.908	0.442
Philippins	3.96	6.96	0.570	0.314	1.21	0.259
Portugal	**12.2**	**8.15**	**1.49**	**1.11**	**1.22**	**0.907**
Korea	6.20	10.6	0.586	0.484	1.65	0.294
Rwanda	0.843	6.76	0.125	0.090	0.832	0.109
Senegal	3.27	6.49	0.505	0.296	1.09	0.270
Sierra Leona	1.67	7.72	0.217	0.139	1.17	0.118
South Africa	4.97	5.98	0.832	0.446	0.902	0.494
**Spain**	**13.7**	**7.68**	**1.78**	**1.25**	**1.11**	**1.13**
Sri Lanka	2.29	6.96	0.329	0.228	1.26	0.181
Swaziland	3.81	6.81	0.560	0.384	0.810	0.474
**Sweden**	**15.5**	**5.53**	**2.81**	**1.60**	**0.810**	**1.97**
Togo	1.33	6.04	0.220	0.104	0.941	0.111
Trinidad	6.80	5.84	1.17	0.677	0.875	0.774
Uganda	2.15	9.13	0.236	0.206	0.929	0.222
**United Arab emirates**	**11.5**	**9.04**	**1.27**	**1.09**	**1.35**	**0.811**
**United Kingdom**	**12.6**	**6.30**	**2.00**	**1.27**	**0.922**	**1.37**
Tanzania	2.04	8.12	0.251	0.194	1.05	0.185
**USA**	**16.0**	**6.87**	**2.33**	**1.61**	**0.974**	**1.65**
Viet Nam	2.65	7.38	0.359	0.199	1.37	0.145
Yemen	2.31	5.92	0.391	0.216	0.879	0.246
Zambia	1.76	5.60	0.315	0.142	0.788	0.180
Zimbabwe	2.07	5.09	0.407	0.209	0.703	0.297

Table S2. Average GDP during the period 1980-2009 (coinciding with the growth period of the 1980s cohort), average daily dietary energy consumption per capita (2006-2008), men height average by country (1980s cohort), average percent of urban population during the period 1990-2009, average human development index (HDI) (1990-2015) and percentage of low weight infants at birth (less than 2500 g) (1980s cohort).

Country	GDP ($)	Calories intake	Height (cm)	Urban population (pct)	HDI	% low weight infants at birth
Bangladesh	348.6	2270	162.40	21.766	0.427	30
**Belgium**	24777	3690	178.80	96.656	0.84	8
Bolivia	933.2	2100	164.20	57.603	0.57	9
Botswana	4395	2230	176.10	43.112	0.573	10
Brazil	3729	3120	171.70	76.426	0.645	10
Burkina	281	2233	172.80	15.993	0.378	19
Cabo Verde	3323	2530	169.00	45.689	0.572	13
Cambodia	397	2180	162.50	16.590	0.391	11
Cameroon	776	2240	171.00	42.076	0.44	11
**Canada**	23609	3530	179.60	77.989	0.858	6
Chad	357	2010	173.30	20.944	0.332	17
Chile	9385	2960	173.10	84.716	0.731	5
China	880	2990	171.50	31.646	0.544	6
Colombia	2327	2690	170.60	69.578	0.625	9
Congo	1184	2570	168.40	56.124	0.512	17
**Denmark**	32112	3410	183.20	85.011	0.88	5
Djibouti	2075	1981	168.90	75.834	0.365	18
Egypt	954	3160	171.30	43.265	0.584	12
**Finland**	25550	3220	178.20	79.641	0.82	4

Gabon	5151	2710	169.70	73.040	0.626	14
**Germany**	25273	3540	180.50	73.174	0.828	7
Ghana	414	2900	170.00	39.910	0.471	11
**Greece**	13442	3710	177.30	72.239	0.779	8
Guatemala	1559	2150	160.80	42.959	0.518	13
Guinea	256	2550	169.50	29.394	0.526	12
Guyana	1033	2740	170.50	29.215	0.572	22
Haiti	434	1850	171.50	32.824	0.43	21
Honduras	988	2610	164.70	42.784	0.532	9
India	471	2360	164.40	26.606	0.462	30
Indonesia	940	2550	164.00	35.623	0.569	9
Iran	2848	3050	172.40	59.949	0.616	7
**Ireland**	25338	3590	176.60	58.112	0.816	6
**Italy**	20173	3650	174.50	67.131	0.798	6
Jamaica	4796	2642	173.60	50.477	0.686	9
**Japan**	29923	2800	171.70	79.799	0.836	8
Jordan	4560	3000	170.30	74.833	0.664	10
Kenya	795	2030	171.20	18.555	0.46	11
Lesotho	456	2460	167.40	16.848	0.468	14
Liberia	207	2200	167.30	45.751	0.359	14
Madagascar	266	2130	162.90	25.018	0.456	14
Malawi	195	2150	166.20	12.729	0.312	16
Mali	314	2590	172.20	25.935	0.273	23
Mexico	4640	3260	167.80	72.626	0.673	9
Morocco	1368	3260	170.30	50.198	0.493	11
Mozambique	288	2070	165.20	25.020	0.259	14
Myanmar	1255	2166	161.30	26.134	0.388	15
Namibia	2457	2360	171.30	30.701	0.567	14
Nepal	227	2340	163.00	11.017	0.417	21
**Netherlands**	26754	3000	182.70	73.556	0.877	4
Nicaragua	932	2420	166.40	53.421	0.53	12
Niger	221	2390	170.70	15.618	0.236	17
Nigeria	470	2710	169.50	31.984	0.493	14
Norway	39135	3450	179.70	74.080	0.883	5
Panama	7589	2450	170.00	57.732	0.682	10
Peru	1900.5	2410	164.20	70.732	0.645	11
Philippins	2140	2580	165.60	45.857	0.605	20
Portugal	11091	3580	172.10	51.014	0.765	8
Korea	11201	3040	171.90	74.183	0.622	23
Rwanda	262	2090	167.20	11.112	0.288	9
Senegal	598	2280	174.30	39.328	0.374	18
Sierra Leona	631	2120	165.80	34.222	0.28	18
South Africa	24243	3000	168.10	54.368	0.626	15
**Spain**	15589	3260	175.60	75.678	0.792	6
Sri Lanka	9760	2370	165.80	18.513	0.649	22
Swaziland	1538	2290	170.20	22.028	0.516	9
**Sweden**	28825	3110	180.40	83.705	0.856	4
Togo	306	2150	167.70	30.618	0.414	15
Trinidad	9888	2771	174.40	9.755	0.695	23
Uganda	281	2220	169.30	11.203	0.351	12
**United Arab emirates**	69799	3170	169.10	80.288	0.762	15
**United Kingdom**	23344	3450	176.80	78.837	0.819	8
Tanzania	282	2020	166.90	20.535	0.381	13
**USA**	30453	3750	179.00	77.101	0.871	8
Viet Nam	1224	2780	160.00	22.851	0.525	9
Yemen	1300	2050	167.60	23.465	0.421	32
Zambia	474.7	1880	168.20	37.817	0.418	12
Zimbabwe	562.7	2210	171.00	30.193	0.464	11

Table S3. Parameter estimates of the levels model, for the 1960s, 1970s and 1980s cohorts. The second column indicates the probability that a coefficient is different from zero. The results are based on an MCMC using Zellner's g-prior (Zellner 1986) and using 40,000 draws, with the first 20,000 discarded as burn-in. To assess convergence of the posterior chain, we used Geweke's test statistic (Geweke 1992), which indicated convergence with a value of 1.02.

		(i) Countries with animal N intake/plant N intake + animal P intake/plant P intake < 2							
Coefficient	p!=0	EV	SD	Model 1	Model 2	Model 3	Model 4	Model 5
	(a)	(b)	(c)	(d)	(e)	(f)	(g)	(h)
Intercept	100.0	-0.4111	0.0024	-0.4111	-0.4111	-0.4111	-0.4111	-0.4111
GDP	15.5	0.0156	0.0121					0.1041
GDP growth	7.9	0.0011	0.0040					
per capita Calories	18.1	0.0221	0.0154			0.1167		
per capita P intake	8.1	0.0041	0.0073					
per capita N intake	14.6	0.0230	0.0176				0.1620	
N/P from animals	9.7	0.0037	0.0049					
N/P from plants	26.2	-0.0218	0.0125		-0.0762			
per capita N/P	99.8	0.2906	0.0614	0.2979	0.3146	0.2640	0.2534	0.2895
pct. Urban population	9.1	-0.0041	0.0065					
1970 cohort dummy	7.6	-0.0004	0.0041					
1980 cohort dummy	9.5	-0.0025	0.0052					
		(ii) Countries with animal N intake/plant N intake + animal P intake/plant P intake > 2						
GDP	95.7	0.2967	0.0756	0.3534	0.2722	0.3093	0.3050	0.2888
GDP growth	22.8	-0.0451	0.0270					
per capita Calories	20.9	0.0400	0.0338			0.2319		
per capita P intake	35.3	0.0961	0.0453		0.2316			
per capita N intake	14.7	0.0382	0.0253					0.2365
N/P from animals	81.1	-0.3459	0.1147	-0.4205	-0.3766	-0.4458	-0.4911	-0.4027
N/P from plants	24.5	-0.0907	0.0520					
per capita N/P	99.5	0.5075	0.1029	0.5114	0.5469	0.4871	0.5018	0.4530
pct. Urban population	19.0	0.0296	0.0215				0.1721	
1970 cohort dummy	21.1	0.0301	0.0186					
1980 cohort dummy	11.9	0.0045	0.0141					
nVar				5	7	7	7	7
BIC				-335.6892	-341.8896	-342.2002	-342.2842	-342.3470
post. prob.				0.2500	0.0801	0.0587	0.0540	0.0507
n				158	158	158	158	158

Table S4. Parameter estimates of the differences in cohorts model, for the 1960s, 1970s and 1980s cohorts. The second column indicates the probability that a coefficient is different from zero. The results are based on an MCMC using Zellner's g-prior (Zellner 1986) and using 40,000 draws, with the first 20,000 discarded as burn-in. To assess convergence of the posterior chain, we used Geweke's test statistic (Geweke 1992), which indicated convergence with a value of 1.02.

	p!=0	EV	SD	Model 1	Model 2	Model 3	Model 4	Model 5
	(a)	(b)	(c)	(d)	(e)	(f)	(g)	(h)
P intake	20.3	0.0192	0.0943					
N intake	23.4	0.0495	0.1317				0.2066	
Diff. GDP	60.7	0.1762	0.1699	0.2591	0.2643	0.3163		0.3938
Diff. GDP growth	39.7	0.0738	0.1084		0.2225			0.1827
Diff. calories	7.8	0.0020	0.0297					
Diff. P intake	74.6	0.4274	0.2920	0.6360	0.5837	0.5626	0.6346	0.5321
Diff. N intake	35.9	0.1077	0.1714					
Diff. P animals/plants	25.5	0.0366	0.0898					0.1946
Diff. N animals/plants	54.9	0.1191	0.1305	0.2298	0.2196		0.2414	
Diff. N/P intake	89.8	0.4848	0.2452	0.6556	0.6111	0.6893	0.6100	0.6506
Diff. urban population	14.0	-0.0063	0.0379					
1970-1980 dummy	45.3	-0.0931	0.1211	-0.2373		-0.2122	-0.2263	
nVar				6	6	5	6	6
BIC				-238.5341	-238.9414	-239.7897	-239.7931	-239.8170
post. prob.				0.0741	0.0526	0.0154	0.0226	0.0134
n				108	108	108	108	108

Bayesian model averaging

The core idea of Bayesian model averaging is to address the model uncertainty over the model space, in terms of choice of explanatory variables, as well as the estimation of the parameters of interest and the size of the model. As established in the pioneering work of Jeffreys (1961), Bayesian model averaging is a practical application of Bayes' rule, by aiming to carry out inference using weighted averaged posterior distributions across alternative models in the model space M. M contains all the potential model combinations formed by the covariates. Given k covariates the full combination would be 2^k potential models, which is the cardinality of M.

Let us denote a particular model $M_m \in M$ (for $m = 1, \dots, 2^k$). We can characterize this model by its parameter vector $\boldsymbol{\beta} = [\beta_1, \dots, \beta_k]$. The posterior density for the parameter vector $\boldsymbol{\beta}$, conditional on the model M_m can be expressed as:

$$(1) \qquad p(\boldsymbol{\beta}|M_m, D) = \frac{p(D|\boldsymbol{\beta}, M_m)p(\boldsymbol{\beta}|M_m)}{p(D|M_m)}$$

where D denotes the data, the prior of $\boldsymbol{\beta}$ is denoted by $p(\boldsymbol{\beta}|M_m)$ and $p(D|\boldsymbol{\beta}, M_m)$ denotes the likelihood. The most frequently used prior for $\boldsymbol{\beta}$ is the g-prior specification by Zellner (1986), since it leads to convenient analytical solutions and enables fast computation of model posteriors.

The candidate models M_m are treated as random in Bayesian model averaging, thus the posterior model probability $p(M_m|D)$ can be expressed by

$$(2) \qquad p(M_m|D) = \frac{p(D|M_m)p(M_m)}{p(D)} \propto p(D|M_m)p(M_m)$$

where $p(M_m)$ denotes the model prior. A natural, fairly agnostic choice for such a prior would be a uniform prior specification in the form of $p(D|M_m) = 2^{-q}$. This treats all candidate models as equally likely a priori. $p(D|M_m)$ denotes the marginal likelihood of model M_m and is specified as:

$$(3) \qquad p(D|M_m) = \int_0^\infty \int_{-\infty}^\infty p(D|\boldsymbol{\beta}, M_m)\, p(\boldsymbol{\beta}|M_m)\, d\boldsymbol{\beta}\, d\sigma^2$$

where the use of the g-prior allows for analytical integration over all the parameters. Using the law of total probability, Bayesian model averaging consists of producing weighted averaged parameter estimates with the posterior model probabilities $p(M_m|D)$ as weights, which are proportional the Bayesian Information Criterion (BIC), see Raftery (1995). Thus, we can specify the posterior density of the parameters as

$$p(\boldsymbol{\beta}|D) = \sum_{m=1}^{2^k} p(M_m|D)p(\boldsymbol{\beta}|M_m, D). \quad (4)$$

If k is large, the number of terms in this equation can quickly rise in such a way that exhaustive summation is infeasible. To circumvent this problem, Markov Chain Monte Carlo techniques can be used, in order to approximate the posterior density. For an overview see Madigan et al. (1995).

References

Geweke, J. (1992) Evaluating the accuracy of sampling-based approaches to the calculation of posterior moments. In: Bernardo, J.M., Bergerm J.O., Dawid, A.P. and Smith, A.F.M., (eds.) Bayesian Statistics. Oxford University Press, Oxford, 4th edition, 167–193.

Jeffreys, H. (1961) Theory of probability. 3rd edition. Clarendon Press, Oxford.

Leamer, E.E. (1978), Specification Searches: Ad Hoc Inference with Nonexperei- mental Data. Wiley & Sons, New York.

Madigan, D., York, J., Allard, D. (1995) Bayesian graphical models for discrete data. International Statistical Review 63(2), 215–232.

Raftery, A.E. (1995) Bayesian model selection in social research. Sociological Methodology 25, 111–163.

Sala-i-Martin, X., Doppelhofer, G., Miller, R. (2004) Determinants of long-term growth: A Bayesian Averaging of Classical Estimates (BACE) approach. American Economic Review 94(4), 813-835.

Zellner, A. (1986) Bayesian Inference and Decision Techniques: Essays in Honor of Bruno de Finetti, chapter, On Assessing Prior Distributions and Bayesian Regression Analysis with g-Prior Distributions. North-Holland, Amsterdam-

Figure S1. . Relationships of mean male height with the average annual daily calories intake (a), GDP (b), HDI (c) and % of low weight infants at birth (d) in the corresponding country and cohort. See the caption for Figure 1 for the country abbreviations. Men born in the 1960s, 1970s and 1980s are identified by 6, 7 and 8 in the symbols, respectively. The countries with higher N and P intake from animal than vegetal products are indicated in red (also red arrow for the mean), and the countries with higher N and P intake from vegetal products are indicated in blue (also the arrow for the mean). Different letters on the axes indicate significant differences (*P*<0.05).

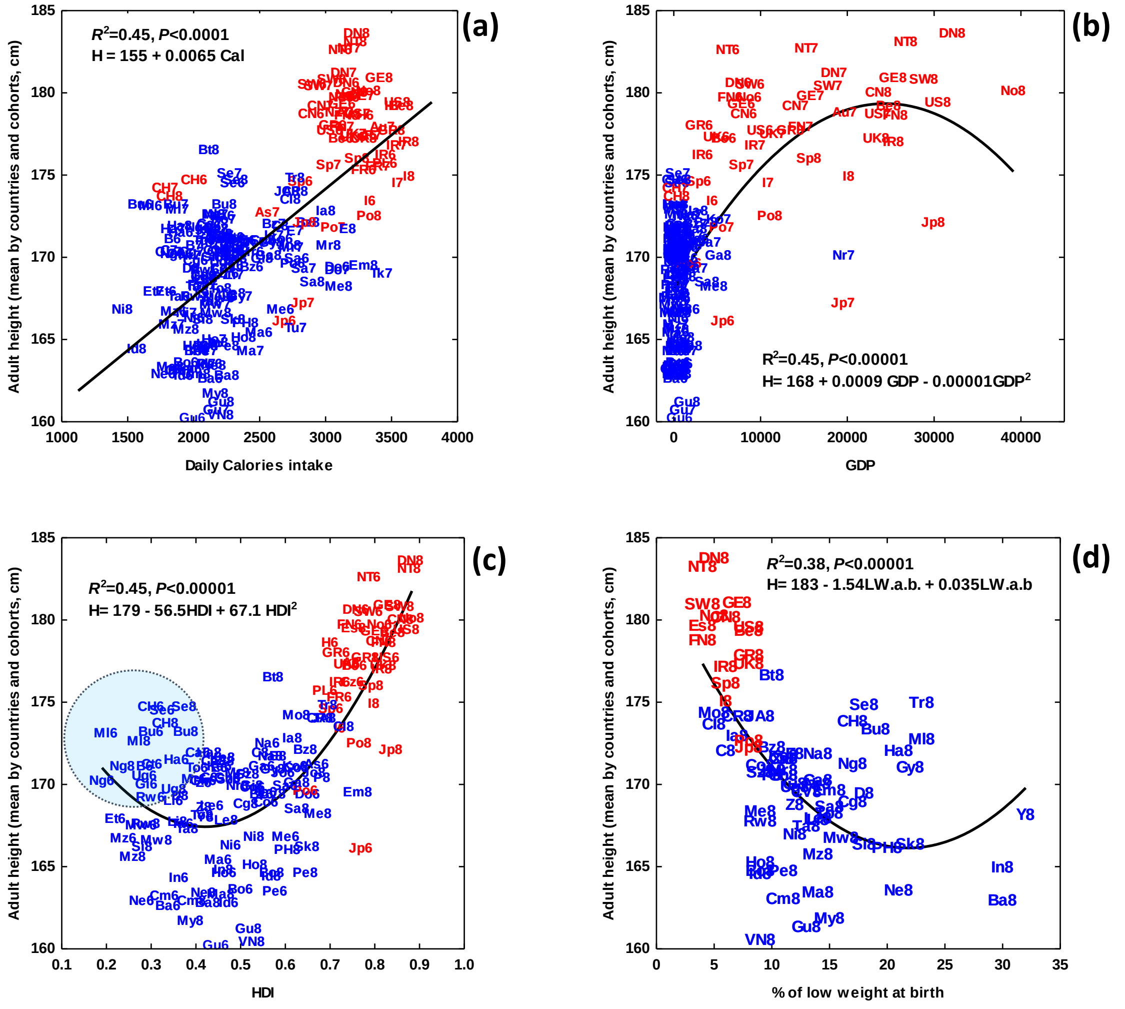

Figure S2. Structural model with differences (men cohort of 1980s) between all pairwise comparisons of the 80 countries studied of GDP, HDI, per capita annual N, P and N:P ratio intake, daily per capita calories intake and % of low weight infants at birth as exogenous variables and the corresponding differences in adult men height as endogenous variable.

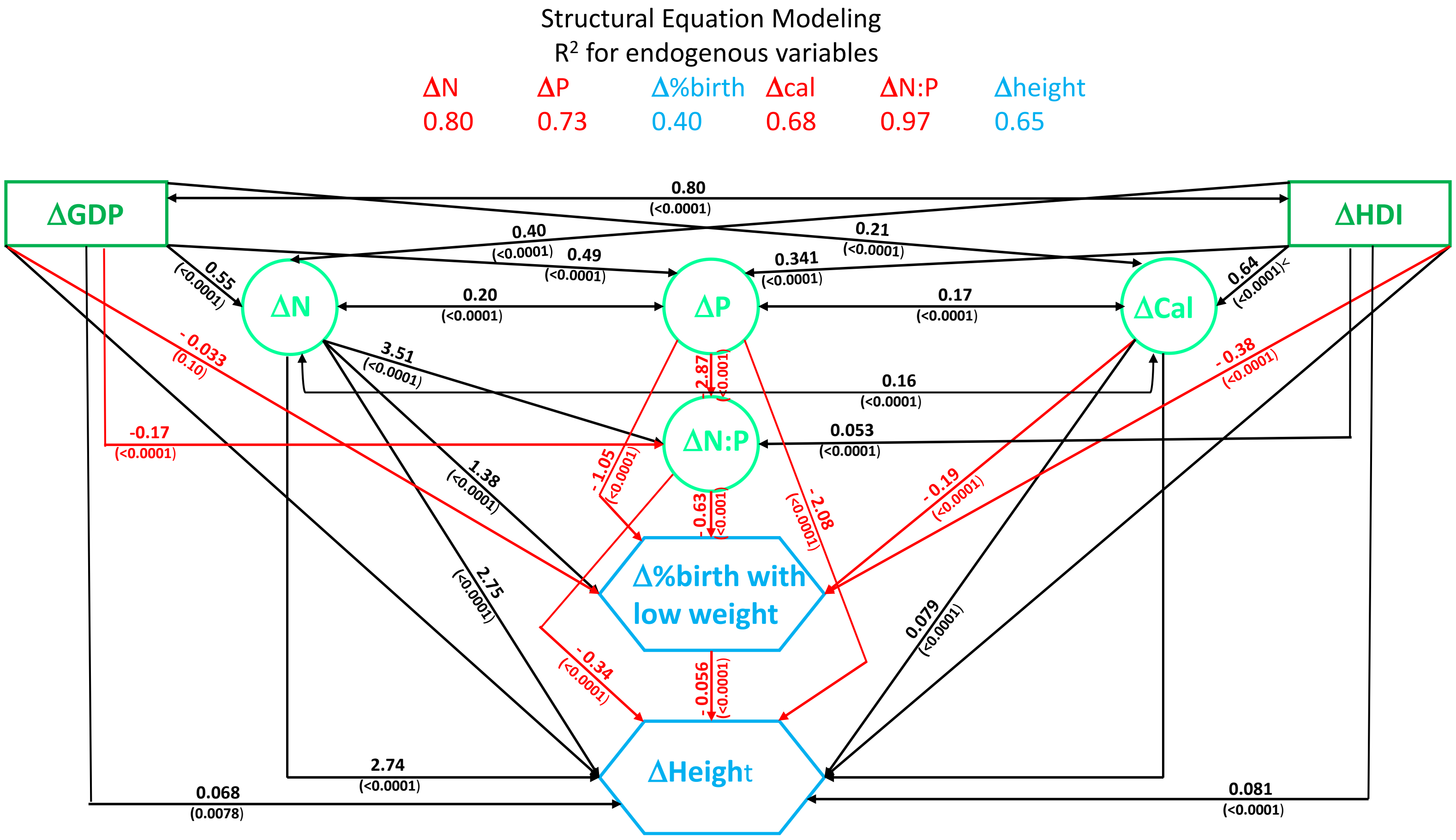

Additional Bayesian analyses also considering possible roles of % of low weight infants at birth and HDI

We modeled human height in absolute levels and across the cohorts with a Bayesian model averaging approach. For this purpose we had observations from 75 countries. The average male human height in a country i in cohort t was modeled as:

$$height_{it} = \sum_{k=1}^{2} \left[\beta_1 + \propto_t + \beta_2 N_{it} + \beta_3 P_{it} + \beta_4 GDP_{it} + \beta_5 GDPg_{it} + \beta_6 Cal_{it} + \beta_7 N_{it} + \beta_8 P_{it} + \beta_9 \frac{N}{P}_{it} + \beta_{10} P_{animal}/P_{vegetables}{}_{it} + \beta_{11} N_{animal}/N_{vegetables}{}_{it} + \beta_{12} Urban_{it} + \beta_{13} w_{it} + \beta_{14} HDI_{it} \right] I_{(\omega_{itk}=1)} + \varepsilon_{it}$$

where $height_{it}$ denotes the height of the t-th cohort in country i. As in the previous equation, β_1 is the intercept, $\propto_t$ a period specific dummy, N_{it} and P_{it} are the levels of N and P intake per capita in cohort t GDP_{it}, $GDPg_{it}$, Cal_{it}, and $Urban_{it}$ denote the level of GDP, GDP growth, calories and percentage of urban population, respectively. w_{it} and HDI_{it} denote the percentage of low birth-weights and the HDI in country i in cohort t. The ratios of N and P intake, as well as N and P intake from animals in proportion to plants are denoted by $P_{animal}/P_{vegetables}{}_{it}$, and $N_{animal}/N_{vegetables}{}_{it}$, respectively. β_2 to β_{14} are the corresponding coefficients, and ε_{it} is the i.i.d. normally distributed error term with zero mean and σ^2 variance. $I_{(\cdot)}$ denotes an indicator function, which takes on the value of one if the condition $\omega_{itk} = 1$ is fulfilled and is zero otherwise. ω_{it1} takes on the value of one if the sum of the N

intake from animals per the N intake from plants plus the P intake from animals per the P intake from plants exceeds two and is zero otherwise. In this fashion we can model the impacts of both groups in one joint model, with a joint variance function.

Table S5. Parameter estimates of the levels model, for the 1980s cohort. The second column indicates the probability that a coefficient is different from zero. The results are based on an MCMC using Zellner's g-prior (Zellner 1986) and using 40,000 draws, with the first 20,000 discarded as burn-in. To assess convergence of the posterior chain, we used Geweke's test statistic (Geweke 1992), which indicated convergence with a value of 1.07.

	p!=0	EV	SD	Model 1	Model 2	Model 3	Model 4	Model 5
Intercept	100.0	0.0000	0.0046	0.0000	0.0000	0.0000	0.0000	0.0000
GDP	22.4	0.0543	0.0281				0.1557	0.1528
GDP growth	8.7	-0.0013	0.0069					
per capita Calories	12.3	0.0172	0.0184					
per capita P intake	9.7	0.0341	0.0103					
per capita N intake	81.6	0.4125	0.0893	0.5402	0.5114	0.5147	0.4051	0.3790
N/P from animals	40.1	-0.0764	0.0411		-0.1550			-0.1542
N/P from plants	26.6	-0.0194	0.0311			-0.1366		
per capita N/P	97.0	0.3239	0.0867	0.3292	0.3106	0.3188	0.3311	0.3124
pct. Urban population	8.5	0.0011	0.0090					
pct. Low birth weight	11.1	-0.0081	0.0104					
HDI	8.8	-0.0020	0.0122					
nVar				3	4	4	4	5
BIC				-139.3510	-143.8279	-144.3053	-145.4511	-149.9135

post. prob.	0.1702	0.1451	0.0900	0.0286	0.0248
n	75	75	75	75	75

Figure S3. Posterior densities of coefficient impacts for the differences in the 1960s, 1970s and 1980s cohorts, ordered by posterior inclusion probability. A high density mass at zero (signified by the vertical dashed line) corresponds to a high probability of the coefficient being excluded from the model. The bold, continuous colored lines denote the median posterior impact of the coefficient, conditional on its posterior inclusion probability. The dashed colored lines depict +/- two posterior standard deviations, conditional on the coefficient being included in the model.

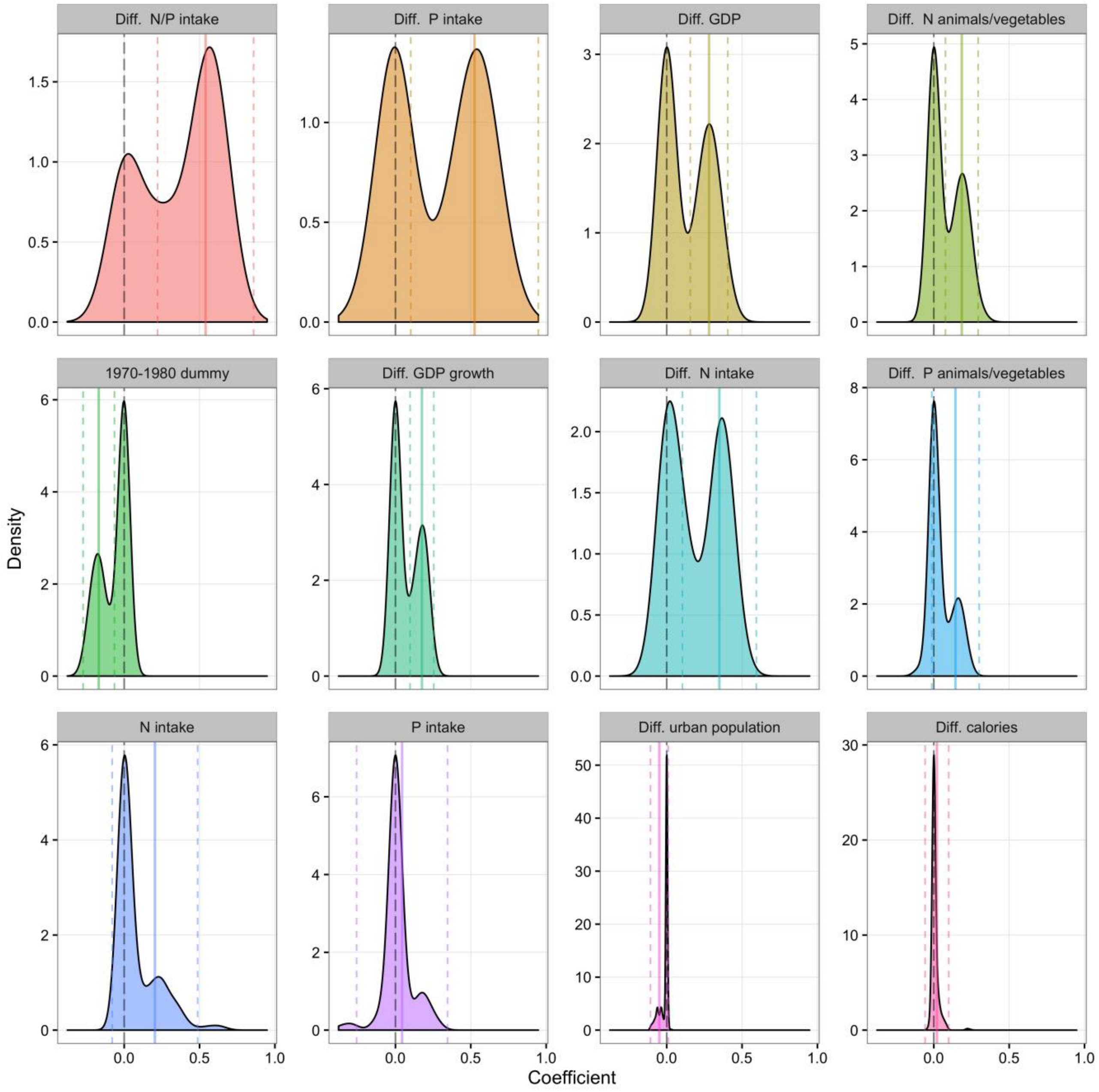

Diff. N/P intake
Diff. P intake
Diff. GDP
Diff. N animals/vegetables
1970-1980 dummy
Diff. GDP growth
Diff. N intake
Diff. P animals/vegetables
N intake
P intake
Diff. urban population
Diff. calories
Density
Coefficient
